# A novel approach to investigate tissue-specific trinucleotide repeat instability

**DOI:** 10.1186/1752-0509-4-29

**Published:** 2010-03-19

**Authors:** Jong-Min Lee, Jie Zhang, Andrew I Su, John R Walker, Tim Wiltshire, Kihwa Kang, Ella Dragileva, Tammy Gillis, Edith T Lopez, Marie-Josee Boily, Michel Cyr, Isaac Kohane, James F Gusella, Marcy E MacDonald, Vanessa C Wheeler

**Affiliations:** 1 Center for Human Genetic Research, Massachusetts General Hospital, Boston, MA, USA; 2 The Genomics Institute of the Novartis Research Foundation, San Diego, CA, USA; 3 Department of Genetics and Complex Diseases, Harvard School of Public Health, Boston, MA, USA; 4 Neuroscience Research Group, University of Quebec at Trois-Rivieres, Trois-Rivieres, Quebec, Canada; 5 Children's Hospital Informatics program, Children's Hospital, Boston, MA, USA; 6 Current address: Obesity and Metabolic Diseases, Regeneron Pharmaceutical, Inc., Tarrytown, NY, USA

## Abstract

**Background:**

In Huntington's disease (HD), an expanded CAG repeat produces characteristic striatal neurodegeneration. Interestingly, the *HD *CAG repeat, whose length determines age at onset, undergoes tissue-specific somatic instability, predominant in the striatum, suggesting that tissue-specific CAG length changes could modify the disease process. Therefore, understanding the mechanisms underlying the tissue specificity of somatic instability may provide novel routes to therapies. However progress in this area has been hampered by the lack of sensitive high-throughput instability quantification methods and global approaches to identify the underlying factors.

**Results:**

Here we describe a novel approach to gain insight into the factors responsible for the tissue specificity of somatic instability. Using accurate genetic knock-in mouse models of HD, we developed a reliable, high-throughput method to quantify tissue *HD *CAG repeat instability and integrated this with genome-wide bioinformatic approaches. Using tissue instability quantified in 16 tissues as a phenotype and tissue microarray gene expression as a predictor, we built a mathematical model and identified a gene expression signature that accurately predicted tissue instability. Using the predictive ability of this signature we found that somatic instability was not a consequence of pathogenesis. In support of this, genetic crosses with models of accelerated neuropathology failed to induce somatic instability. In addition, we searched for genes and pathways that correlated with tissue instability. We found that expression levels of DNA repair genes did not explain the tissue specificity of somatic instability. Instead, our data implicate other pathways, particularly cell cycle, metabolism and neurotransmitter pathways, acting in combination to generate tissue-specific patterns of instability.

**Conclusion:**

Our study clearly demonstrates that multiple tissue factors reflect the level of somatic instability in different tissues. In addition, our quantitative, genome-wide approach is readily applicable to high-throughput assays and opens the door to widespread applications with the potential to accelerate the discovery of drugs that alter tissue instability.

## Background

Expansions of trinucleotide repeat sequences over certain thresholds cause more than 30 human diseases including Huntington's disease (HD), a number of spinocerebellar ataxias (SCAs), myotonic dystrophy 1 (DM1), and fragile X syndrome. Interestingly, expanded trinucleotide repeat sequences undergo progressive, expansion-biased tissue-specific somatic instability [1-6]. As the severity of these disorders is highly dependent on repeat length, somatic instability in tissues that are the pathogenic targets is predicted to contribute to disease. Notably, in HD, striking somatic expansion of the *HD *CAG repeat occurs in the striatum and cortex, brain regions that are major targets of the pathogenic process. Furthermore, studies both in HD patients and in a knock-in mouse model of HD provide compelling evidence indicating that somatic expansion in these brain regions accelerates the ongoing pathogenic process [7-9]. Therefore, understanding the mechanisms underlying tissue-specific somatic instability in HD may provide novel routes to therapies.

Somatic instability is critically dependent on DNA repair genes and is also influenced by *cis*-factors [7,8,10-16]. However, it is unknown what determines its tissue specificity. It has been proposed that the expression levels of DNA repair genes and/or the pathogenic process itself may underlie tissue patterns of instability [5]. Given that somatic *HD *CAG instability occurs in many tissues to varying extents [3,6,17], we reasoned firstly, that tissue specificity may governed by many factors, and secondly, that studying a large cross-section of tissues with different instabilities would provide the most information concerning the major factors underlying tissue instability patterns. Therefore, in order to gain insight into the factors that govern the tissue specificity CAG instability in HD, we have taken quantitative, global and unbiased approaches.

Using accurate genetic knock-in mouse models of HD [6,18] that exhibit similar tissue-specific patterns of somatic instability to those seen in HD patients [3,6], we developed a novel instability quantification method that is sensitive and applicable to high-throughput assays. We then integrated this methodology with unbiased and global bioinformatic approaches to identify a gene expression "signature" and biological pathways that correlate with tissue instability. Using these methods we have, a) tested the role played by factors previously proposed to contribute to the tissue specificity of somatic instability, and b) uncovered novel pathways that may be important in determining the tissue specificity of instability in HD.

## Results

### Instability quantification

**Figure 1:**
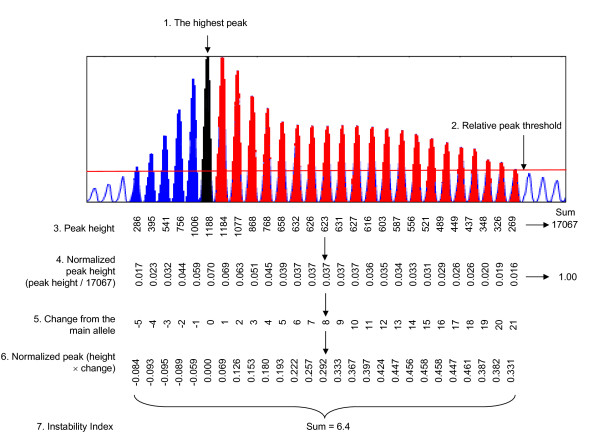
**Instability index determination using a relative peak height threshold**. To quantify the levels of instability from GeneMapper traces peak height was used to determine a relative threshold of 20%. Peaks falling below his threshold were excluded from analysis. Peak heights normalized to the total of all peak heights were multiplied by the change in CAG length of each peak relative to the highest peak in tail (main allele). These values were summed to generate an instability index. Striatum analysis is shown as an example (*Hdh*^*Q*111/+^, 5 months, 100 ng genomic DNA). Open, blue, black, and red peaks represent background, contracted alleles, main allele from tail analysis of same mouse, and expanded alleles, respectively.

Previous methods for determining instability following PCR amplification of repeats from 'bulk' genomic DNA have either been qualitative, or have failed to adequately account for amplification efficiencies that differ between stable and unstable tissues. In contrast, quantitative small pool-PCR (SP-PCR) methods [19] are extremely labor-intensive and impractical for high-throughput analyses. In order to facilitate high-throughput, global analyses of somatic instability we therefore first developed a novel method for quantifying CAG repeat sizes from 'bulk' genomic DNA. PCR amplification of trinucleotide repeats generates multiple PCR products, viewed using GeneMapper software as a cluster of peaks differing by a single CAG repeat unit (Figure 1). Distinguishing signal peaks from noise peaks is critical for the accurate measurement of instability. In typical GeneMapper traces of PCR-amplified trinucleotide repeats, there is no clear boundary between signal and noise, making defining noise peaks (or background signals) extremely difficult. To solve this problem, we developed a novel background correction method (namely, relative peak height threshold), where 20% of the height of the highest peak was set as the threshold for each analysis. For stringent analyses, peaks with heights lower than this threshold level were excluded from quantification. We used a conservative threshold factor (20%) in this study as this detects peaks with good signal intensity (i.e. over 100), and is more resistant to amplification variation than lower thresholds (i.e. 10%). However, if peak signals are strong enough, a lower threshold (10%, 5%) will provide more sensitive quantification.

**Figure 2:**
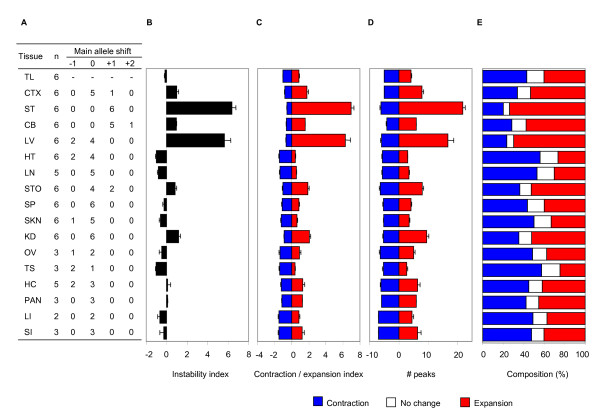
**Reproducibility of instability quantification methods**. Instability indices were determined from 100 ng genomic DNA isolated from 17 tissues of 2-6 *Hdh*^*Q*111/+ ^mice at 5 months of age. (A) The table shows replicate number of mice for each tissue (n) and numbers of samples where the highest peak was shifted from the main allele (tail's highest peak). Zero indicates no major allele shift and +1 indicates one CAG unit increase. B. Instability index. (C, D, E) The relative peak height correction method was applied to different methods of quantification such as: a contraction and expansion index (C), the number of contracted/expanded peaks (D) and the relative composition of contracted/unchanged/expanded peaks (E). Data bars represent mean ± SE. TL, tail; CTX, cortex; ST, striatum; CB, cerebellum; LV, liver; HT, heart; LN, lung; STO, stomach; SP, spleen, SKN, skin; KD, kidney; OV, ovary; TS, testis; HC, hippocampus; PAN, pancreas; LI, large intestine; and SI, small intestine.

Figure 1 illustrates the procedure for instability quantification. This is outlined as follows: 1) the highest peak (arrow) in each analysis was identified; 2) 20% (threshold factor) of the height of the highest peak was set as a relative peak height threshold (red horizontal line); 3) for background correction, peaks with heights less than the threshold were excluded; 4) normalized peak heights were calculated by dividing the peak height of each peak by the sum of the heights of all signal peaks; 5) the change in CAG length of each peak was deduced from the constitutive CAG length of the mouse determined by the highest peak in tail analysis (main allele); 6) the normalized peak heights were multiplied by the changes from the main allele; 7) these values were summed to get the instability index. The instability index represents the mean CAG length change from the main allele per cell in a given tissue. Theoretically, symmetrical distribution of contraction and expansion will result in an instability index of zero. However, as instability in *Hdh*^*Q*111 ^mice is expansion-biased and contraction is not highly variable between tissues (see Figure 2), this quantification effectively captures repeat expansion.

### Validation of the relative peak height threshold quantification method

We first determined the reproducibility of our method by quantifying instability index in 17 tissues from 2-6 different *Hdh*^*Q*111/+ ^mice at 5 months of age. As shown in Figure 2, the shift in the highest peak compared to tail (panel A) and the instability index (panel B) were highly reproducible between mice for all tissues tested. Note that the instability indices of stable tissues (i.e. lung, heart, spleen) were negative because stable tissue GeneMapper traces were biased toward contraction likely due to the increased amplification efficiency of shorter CAG alleles. Instability indices of 17 tissues ranged from -1.03 (testis) to 6.37 (striatum).

The relative peak height threshold method can also be applied to different types of instability quantification depending on the focus of the biological question. Thus, after applying the relative peak height threshold, we can determine contraction and expansion indices (Figure 2C), the number of contracted and expanded peaks (Figure 2D) or the relative composition (%) of contracted, expanded and unchanged peaks (Figure 2E). Importantly, these measurements of different aspects of instability may be useful to capture the complexity of tissue instability. In all cases, measurements were reproducible for all tissues across multiple mice. To represent the levels of instability of tissues for further analysis, we used the instability index (Figure 2B).

**Figure 3:**
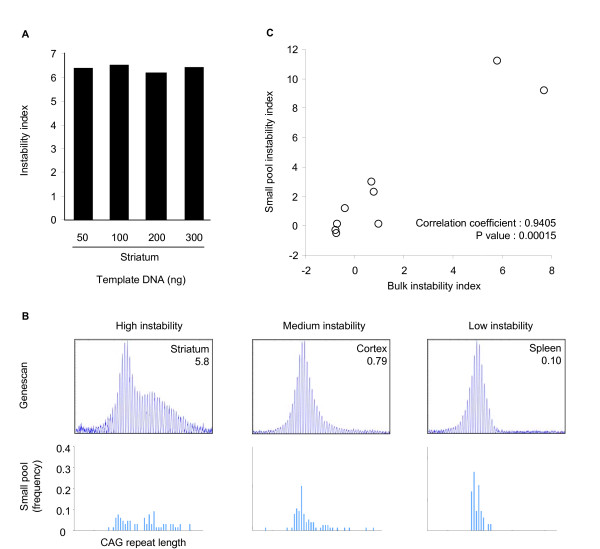
**Evaluation of instability index**. (A) To assess the sensitivity of the instability index to the amount of input DNA we calculated instability indices using varying amounts of template DNA (*Hdh*^*Q*111/^, 5 months, striatum). The coefficient of variation (CV) of the striatal instability index was 2.2%, calculated by dividing the standard deviation of 4 instability index measurements (50, 100, 200, and 300 ng DNA) by the mean instability indices of same 4 measurements. (B) GeneMapper traces and instability indices from bulk DNA (100 ng) and frequency distributions of CAG repeat length obtained using small pool-PCR from striatum, cortex and spleen from an *Hdh*^*Q*111/+ ^mouse at 5 months of age. (C) Instability indices from bulk DNA (100 ng) GeneMapper traces were plotted against small pool instability indices (see Methods section) obtained from 9 tissues (striatum, cortex, cerebellum, liver, lung, stomach, skin, heart, ovary) of an *Hdh*^*Q*111/+ ^mouse at 5 months of age. The two values were highly correlated.

Next, to examine the effect of template DNA amount on instability index, we calculated striatal instability indices using different amounts of template DNA from striatum of an *Hdh*^*Q*111/+ ^mouse at 5 months of age. As shown in Figure 3A, instability indices calculated using the relative peak height method generated consistent instability indices (coefficient of variation, 2.2%) from a wide range of template DNA amounts (50~300 ng).

We then compared instability indices using our relative peak height threshold method to somatic instability quantified using SP-PCR on genomic DNA of tissues from the same mouse (9 tissues, 5 month, *Hdh*^*Q*111/+^). Figure 3B shows examples of tissues exhibiting high, medium and low instability indices, and the corresponding CAG repeat length frequency distributions obtained by SP-PCR. These data indicated that the instability index broadly captured the bulk of the somatic variation detected by SP-PCR, but not the rare large expansions. However, there was a highly significant correlation between the instability index obtained using the bulk DNA method and an instability index quantified from the small pool data (Figure 3C, p value, 0.00015), suggesting that although instability index using bulk DNA may not be sensitive enough to detect rare molecules, it can give a good estimate of overall instability.

Together, our analyses suggest that the instability index, determined from GeneMapper traces of bulk genomic DNA, is a reproducible measurement, relatively insensitive to input DNA amount and well suited for high-throughput analyses where SP-PCR may be impractical.

### Genome-wide identification of an instability-correlated gene expression signature

With the aim of investigating the tissue specificity of somatic instability in a global and unbiased manner we then took a bioinformatics approach. Using 16 different tissues from 5-month *Hdh*^*Q*111/+ ^mice as our training set (Figure 2B, excluding tail), with instability index as a quantitative phenotype, we analyzed mouse tissue gene expression data (Mouse Gene Expression Atlas GSE11339, C57BL/6J, 10 weeks) to identify a gene expression signature that correlated with tissue repeat instability. *Hdh*^*Q*111 ^somatic instability (and therefore instability index) increases over time [6]. We chose 5 months as this represents a time-point at which tissue differences in instability can be readily resolved. Notably, the Gene Expression data is derived from mice that differ in age and genetic background (B6 versus CD1, absence versus presence of *HD *CAG knock-in allele) to the *Hdh*^*Q*111 ^mice in this study. While age and genetic background-related gene expression changes will increase the noise in our system, this broad, tissue-based analysis allows us to pull out major tissue-specific gene expression differences that occur over and above age- and genetic background-related effects.

**Figure 4:**
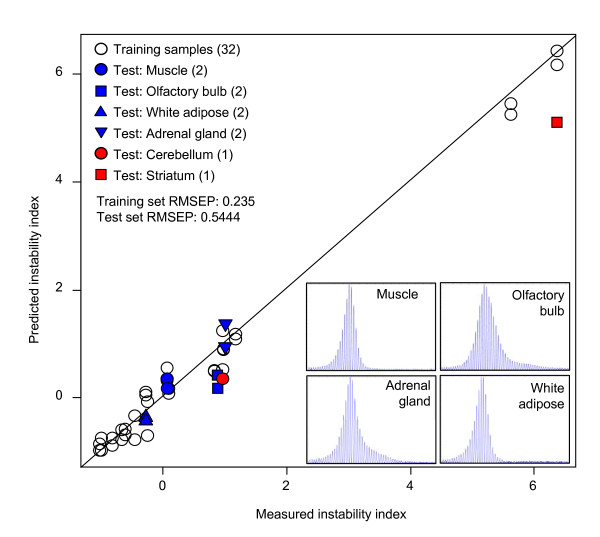
**Instability-correlated gene expression signature and regression modeling**. To identify an instability-correlated gene expression signature, instability index was modeled as a function of gene expression using the mouse Gene Expression Atlas. We calculated the correlation between instability index and expression level, and built regression models by sequentially introducing top n number of the most highly correlated probes with instability index (16 training tissues, 2 gene expression replicates) using partial least square regression (PLSR). The lowest error rate (root mean squared error of prediction, RMSEP) in leave one out cross validation (0.235) was obtained by modeling of the 150 most correlated probes. The predictive power of the model was verified by two independent test sets. Firstly, we determined instability indices of 4 additional tissues, muscle, olfactory bulb, white adipose tissue and adrenal gland (*Hdh*^*Q*111/+^, 5 months, n = 4-6 mice), and compared them with instability indices predicted by the regression model (blue, 2 gene expression replicates). Secondly, we predicted instability indices using independent striatum and cerebellum microarray data (GSE9025, *Hdh*^*Q*111/+^, 5 months, n = 1), and compared them to measured instability indices (red). RMSEP, root mean squared error of prediction.

Thus, we modeled instability index as a function of gene expression using partial least square regression (PLSR) [20]. An instability-correlated gene expression signature was identified by leave-one-out cross validation (LOO CV) of training samples (16 tissues), and the signature, comprised of the 150 most highly correlated probes with tissue instability (Additional file 1), reflected the instability index with a root mean squared error of prediction of 0.235 (Figure 4, training sample RMSEP).

We then confirmed the predictive power of this instability-correlated gene expression signature by comparing measured instability indices with predicted instability indices from our regression model in new independent samples. For this, 1) we measured instability indices of four new independent *Hdh*^*Q*111/+ ^tissues (muscle, olfactory bulb, white adipose tissue and adrenal gland) and compared these with instability indices predicted from the regression model in the same tissues (Figure 4, blue), and 2) we predicted instability indices using independent microarray data from *Hdh*^*Q*111 ^striatum and cerebellum and compared these with measured instability indices (Figure 4, red). As shown in Figure 4, the predicted and measured instability indices matched closely in all cases (test set RMSEP, 0.5444) with a significant correlation (Pearson correlation coefficient, 0.9783; p value, 9.6 × 10^-7^), indicating that instability index can be relatively precisely predicted from the gene expression signature. Furthermore, these data demonstrate that although the model was based on gene expression data and instability index data from mice that differed in age and genetic background, it nevertheless has significant predictive power. This indicates the presence of tissue-specific factors related to instability independent of age and genetic background.

### Tissue instability prediction

Our sensitive quantification method and instability-correlated gene expression signature/regression model is a versatile tool. One of the advantages of our regression model is that the 'propensity' for instability can be predicted when instability can not be directly measured. For example, our approach allowed a prediction of an instability index in 78 different tissues and conditions in the mouse tissue gene expression data set (Table 1), a far greater number than has ever been previously measured, providing a comprehensive view of tissue instability. Interestingly, although most of the tissues (except striatum and liver) were predicted to be relatively stable, some degree of CAG repeat instability was predicted for many tissues in the nervous system.

**Table 1:** Tissue instability index predicted by a PLSR model.

Tissue	Instability index	Tissue	Instability index
dorsal striatum	6.37	cornea	0.29
liver	5.63	common myeloid progenitor	0.29
kidney	1.17	follicular B-cells	0.27
Adrenal gland	1.13	skeletal muscle	0.27
amygdala	1.10	dendritic plasmacytoid B220+	0.26
hypothalamus	1.08	granulo mono progenitor	0.24
retina	0.99	macrophage peri LPS thio 0 hrs	0.22
cerebral cortex	0.98	ciliary bodies	0.17
cerebellum	0.96	mast cells	0.16
mega erythrocyte progenitor	0.96	osteoblast day5	0.14
lens	0.95	bone marrow	0.12
NK cells	0.89	pituitary	0.10
dendritic cells lymphoid CD8a+	0.88	pancreas	0.09
macrophage bone marrow 6 hr LPS	0.85	B-cells marginal zone	0.08
T-cells foxP3+	0.85	hippocampus	0.07
stomach	0.82	lacrimal gland	0.06
dorsal root ganglia	0.75	lymph nodes	0.04
macrophage bone marrow 24 h LPS	0.74	spinal cord	0.03
macrophage bone marrow 2 hr LPS	0.72	mammary gland lact	0.01
macrophage peri LPS thio 1 hrs	0.71	osteoblast day 14	-0.15
cerebral cortex prefrontal	0.69	salivary gland	-0.20
T-cells CD4+	0.63	uterus	-0.24
macrophage peri LPS thio 7 hrs	0.59	mammary gland non-lactating	-0.25
mast cells IgE	0.57	spleen	-0.26
thymocyte DP CD4+CD8+	0.57	intestine small	-0.29
iris	0.57	bone	-0.29
T-cells CD8+	0.49	eyecup	-0.30
osteoclasts	0.48	adipose white	-0.39
macrophage bone marrow 0 hr	0.47	granulocytes mac1+gr1+	-0.39
thymocyte SP CD8+	0.46	osteoblast day 21	-0.39
mast cells IgE+antigen 1 hr	0.43	ovary	-0.47
adipose brown	0.43	bladder	-0.54
dendritic cells myeloid CD8a-	0.41	epidermis	-0.61
mast cells IgE+antigen 6 hr	0.39	intestine large	-0.67
retinal pigment epithelium	0.34	placenta	-0.73
thymocyte SP CD4+	0.33	lung	-0.82
prostate	0.30	heart	-1.01
microglia	0.30	testis	-1.03
olfactory bulb	0.30	umbilical cord	-1.18

The PLSR model built with the instability-correlated gene expression signature from 16 training tissues was used to predict instability index for each of the tissues analyzed in the GNF mouse Gene Expression Atlas. Data represents mean of 2 replicates.

### Pathogenesis and instability

**Figure 5:**
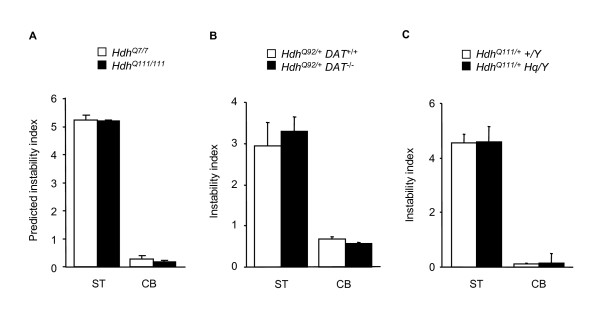
**HD pathogenesis and somatic instability**. (A) We profiled gene expression in striatum and cerebellum in *Hdh*^*Q*111/111 ^and control (*Hdh*^+/+^) mice at 10 weeks of age (GSE19780). Expression values of the 150 instability-correlated probes were used to predict instability indices based on our regression model. Data bars represent mean ± SD (n = 3-5 mice per genotype). (B) Dopamine transporter (DAT) knockout mice were crossed with *Hdh*^*Q*92 ^mice to test if accelerated HD pathogenesis increases instability (8-10 months, n = 3-4 mice per genotype). Instability indices were measured in striatum and cerebellum. Cerebellum was included as a control tissue that does not show accelerated HD pathology. (C) *Hq/+ *mice were crossed with *Hdh*^*Q*111 ^mice to test if pathology in cerebellar granule cells can induce somatic instability (4 months, n = 3 mice per genotype). Instability indices were measured in striatum and cerebellum. Striatum was included as a control tissue that does not show *Hq*-mediated neurodegeneration. In addition, the *Hq *mutation did not increase *HD *CAG instability in cerebellum at either 5 weeks (n = 3-4 per mice genotype) proceeding overt neurodegeneration, or at 7 months (n = 3-4 mice per genotype) when the mice exhibit significant neurodegeneration (data not shown).

We are interested in understanding the factors that contribute to the tissue specificity of *HD *CAG somatic expansion, particularly, why the repeat is so unstable in the striatum. Our instability quantification/bioinformatics approach provides a novel, global and unbiased means of probing these factors. One possibility that could at least in part explain the tissue specificity is that somatic instability occurs as a result of the ongoing HD pathogenic process, as previously hypothesized [21]. We first used our ability to predict instability from gene expression to test this hypothesis. The instability-correlated gene expression signature reflects a cell or tissue state that is associated with instability. If instability occurred as a result of ongoing pathogenesis, one would expect an altered level of instability-correlated gene expression signature in cells expressing mutant huntingtin compared to wild-type cells. Therefore, we performed gene expression profiling on striata and cerebella of 10-week *Hdh*^*Q*111/111 ^mice that exhibit an ongoing pathogenic process and somatic instability in striatum but not in cerebellum [18], and on wild-type *Hdh*^+/+ ^littermates, and predicted instability using the regression model above. Interestingly, as shown in Figure 5A, predicted instability indices were greater in striatum than in cerebellum, but did not distinguish mutant from wild-type striatum. This finding suggested that mutant and wild-type striata have an equal *propensity *for somatic expansion that is unrelated to the *HD *CAG pathogenic process. Although wild-type striata possesses this propensity, the normal *HD *CAG repeat does not actually expand because it does not present a sufficiently long target to be susceptible to the processes that mediate expansion.

To test the prediction that somatic instability does not occur as a consequence of ongoing pathogenesis, we performed two genetic experiments. Since the expanded *Hdh *CAG repeat is both a source of a pathogenic process and a target of instability, it is very difficult to delineate the relationship between the HD pathogenic process and somatic instability. Therefore, we used genetic mouse models in which neurodegenerative processes are modulated or caused by factors independent of the *HD *CAG repeat. We first investigated *Hdh*^*Q*92 ^mice lacking the dopamine transporter (DAT), which show accelerated HD pathogenesis in the striatum [22]. As shown in Figure 5B, striatal instability indices of *Hdh*^*Q*92/+ ^*DAT*^-/- ^and *Hdh*^*Q*92/+^*DAT*^+/+ ^mice were not different, indicating that *HD *CAG instability is not contributed by the disease process. We also tested whether inducing neurodegeneration in the cerebellum, a normally stable tissue, would cause instability in the cerebellum by crossing *Hdh*^*Q*111 ^mice to Harlequin (*Hq*) mice, a model of cerebellar granule cell degeneration [23]. As shown in Figure 5C, *Hdh*^*Q*111/+ ^*Hq/Y *mice and *Hdh*^*Q*111/+ ^*+/Y *control mice exhibited similar low cerebellar instability indices, indicating that neurodegeneration *per se *is insufficient to induce instability.

Taken together, these results support the prediction from our mathematical model, that the *HD *CAG disease process is not responsible for the striatal specificity of *HD *CAG repeat instability, arguing against the sequestration of DNA repair proteins or other factors, as a contributor to somatic instability as previously suggested [21]. Our results are also in agreement with similar levels of instability seen in knock-in and fragment transgenic models of HD that exhibit different rates of inclusion formation [24], and with the observation that striatal instability occurs in SCA1 and DM1, although the striatum is not the target of pathogenesis in these disorders [2,5].

### DNA repair and repeat instability

**Figure 6:**
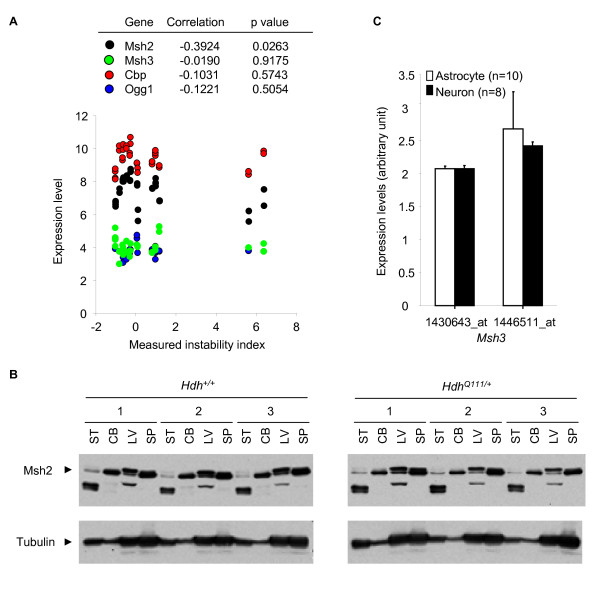
**Candidate DNA repair genes and somatic instability**. (A) Gene expression levels (*Msh2*, *Msh3*, *Cbp*, *Ogg1*) and measured instability indices of 16 training tissues were plotted. Among multiple probes representing the same gene, the probe with the highest expression level in striatum was selected. Expression levels showed insignificant correlations for *Msh3, Cbp *and *Ogg1 *and a negative correlation for *Msh2 *with measured instability indices. (B) Protein levels of Msh2 were measured in 2 unstable (striatum and liver) and 2 stable tissues (cerebellum and spleen) from *Hdh*^*Q*111/+ ^and corresponding control mice (n = 3 mice per genotype, 2 months). Whole cell protein extracts (70 μg) were resolved by SDS-PAGE (6%) and western blots were performed for Msh2 (Santa Cruz Biotechnology) and Tubulin (Cell Signaling). ST, striatum; CB, cerebellum; LV, liver; SP, spleen. (C) Microarray expression levels of *Msh3 *(Affymetrix probe ID, 1430643_at and 1446511_at) in FACS-purified astrocytes and neurons were obtained from the GSE9566 data set. *Msh3 *expression level in astrocytes was not significantly different from that in neurons. Data bar represents mean of log2 expression levels ± SD (n = 8-10).

DNA repair genes, particularly in the mismatch repair pathway, are required for somatic expansion of trinucleotide repeats [7,8,11-16] and have previously been suggested as trans-acting tissue-specific factors responsible for tissue-specific somatic instability [5]. One possibility, therefore, is that DNA repair gene expression levels are correlated with the levels of instability in tissues. Our instability-associated gene expression signature gave us the opportunity to examine if expression levels of DNA repair genes play a role in determining the tissue specificity of instability. Thus, if DNA repair gene expression levels were major determinants of the tissue specificity of somatic instability the expression levels of these genes would be predicted to correlate with instability levels across tissues. Initial examination of the 150 probes comprising our instability-correlated gene expression signature did not highlight an important role for genes involved in DNA repair in general (Additional file 1). To probe these processes further, we examined whether expression levels of specific DNA repair genes (*Msh2*, *Msh3*, *Ogg1 *and *Cbp*), previously shown to play important roles in CAG repeat instability [8,13,14,21,24-26], correlated with instability index measured in 16 tissues. The expression levels of *Msh3*, *Ogg1 *and *Cbp *did not correlate with instability index and *Msh2 *expression level showed a weak negative correlation with instability index (Figure 6A, Additional file 2). In agreement with these findings, and further validating the predictive power of our signature, protein levels of Msh2 (Figure 6B) and Cbp (data not shown) did not correlate with instability index.

Selective neuronal expression of *Msh3 *was recently proposed to contribute to the greater levels of instability in neurons compared to glia [24], and therefore we explored this further. Analyses of gene expression data revealed nearly identical *Msh3 *expression levels in purified neurons and glia (Figure 6C). Together with the lack of correlation between instability index and *Msh3 *expression levels across 16 tissues (Figure 6A, Additional file 2), the data argue against a major role for *Msh3 *expression levels in determining tissue- or cell type-specific instability.

Therefore, our results suggest that although certain DNA repair genes are absolutely critical for somatic instability [7,8,11-16], their expression levels are unlikely to be the primary determinants of tissue specificity. Clearly, posttranscriptional and/or posttranslational regulation of DNA repair genes could still play a tissue-specific role. It would therefore be of further interest to determine whether there is a correlation between DNA repair enzyme activities and tissue instability.

### Genome-wide survey for pathways that correlate with tissue instability

Our findings indicate that neither HD pathogenesis, nor DNA repair gene expression levels is a major determinant of the tissue specificity of somatic instability. We therefore sought to gain insight into the nature of the factors that might influence the tissue specific property of somatic instability. Although, as a group, the genes comprising our signature are highly predictive of instability, on an individual gene basis, they do not have sufficient power to predict instability-related biological pathways. Therefore, as an alternative strategy, we carried out a sensitive, unbiased and global gene set enrichment analysis (GSEA), in which gene expression data is analyzed at a the level of biological pathways rather than individual genes [27]. Confirming the findings above, DNA metabolism pathways were not significantly correlated with instability index (Additional file 3). In contrast, pathways broadly in the cell cycle category were negatively correlated, whereas pathways related to neurotransmitter activity (e.g. adrenoceptor, monoamine, and serotonin) and cellular metabolism (e.g. glycolipid) were positively correlated with tissue instability index (Table 2). Confirming previous predictions, the negative correlation of cell cycle pathways with instability index is consistent with the instability seen in many tissues of the nervous system (Table 1), its occurrence in postmitotic neurons [24,28] and a dissociation of instability and cell division rate [29]. Our results also suggest novel roles for additional pathways (e.g. neurotransmitter and cellular metabolism pathways) in determining the tissue specificity of somatic instability.

**Table 2:** Pathways significantly correlated with the instability index.

Name	Size	NES	P value
**Negative correlation**			

G1 to S cell cycle reactome	150	-1.87	0.0000
Nuclear membrane	208	-1.72	0.0023
Negative regulation of progression through cell cycle	183	-1.64	0.0024
Mitosis	261	-1.82	0.0044
M phase of mitotic cell cycle	262	-1.81	0.0044
Protein kinase inhibitor activity	41	-1.71	0.0046
G1 pathway	68	-1.85	0.0046
Cell cycle pathway	57	-1.85	0.0066
Mitotic cell cycle	427	-1.76	0.0069
Kinase inhibitor activity	42	-1.72	0.0070
Protein amino acid-ribosylation	30	-1.92	0.0071
Eicosanoid synthesis	29	-1.79	0.0072
P53 pathway	43	-1.77	0.0085
Notch pathway	17	-1.70	0.0086
Cell cycle	176	-1.82	0.0087
Integrin mediated cell adhesion	222	-1.73	0.0089
RNA helicase activity	41	-1.78	0.0097
			
**Positive correlation**			

UDP-galactose beta-N-acetylglucosamine beta-1,3-galactosyltransferase activity	21	1.84	0.0000
Adrenoceptor activity	25	1.94	0.0017
Amine receptor activity	47	1.88	0.0018
Beta-1,3-galactosyltransferase activity	25	1.94	0.0020
Mono amine GPCRS	45	1.88	0.0038
Glutamate metabolism	51	1.78	0.0042
Neuromuscular junction development	15	1.76	0.0057
Serotonin receptor activity	22	1.81	0.0057
Oxidoreductase activity, acting on the CH-CH groups of donors, oxygen as acceptor	15	1.72	0.0064

Gene set enrichment analysis was performed using Pearson correlation between expression level and instability index as a ranking metric. Significant gene sets were identified by permutation-based nominal p value (p < 0.01). NES, normalized enrichment score.

It is possible that as striatum is particularly unstable, the highly correlated pathways are simply those that are predominantly present or absent in this tissue, and that the correlation with instability is coincidental. However, pathways significantly up-regulated or down-regulated in striatum compared to cerebellum (data not shown) showed little overlap with those that correlated with instability; for example, the dopamine pathway is strongly up-regulated in striatum, but does not correlate with instability. This suggests that the instability-correlated pathways are directly related to instability rather than simply being striatal-specific.

### Test of prediction from GSEA

**Figure 7:**
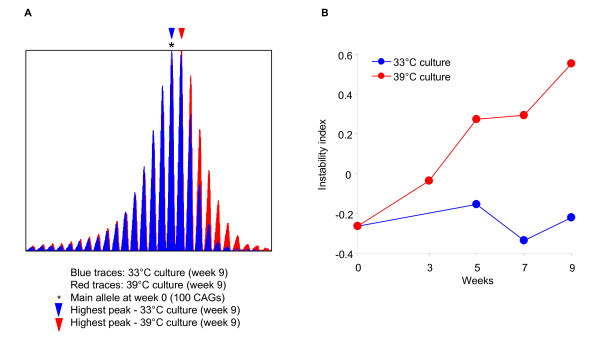
**The effect of cell proliferation on instability index**. (A) ST*Hdh*^*Q*111/+ ^cells were maintained at the restrictive temperature (39°C) (no cell division) for 9 weeks without subculture (red), and control cultures at the permissive temperature (33°C) (actively dividing) were subcultured every week (blue). GeneMapper traces of genomic DNA isolated at 9 weeks are shown. (B) For the time-course study, cultures grown at 39°C and at 33°C were harvested at 0, 3, 5, 7 and 9 weeks, and genomic DNA was analyzed to calculate the instability index. Representative GeneMapper traces and instability indices are shown from three independent experiments.

Instability-correlated pathways may either directly modify instability or may represent cells' secondary responses to instability. To distinguish these alternatives, we asked whether alteration of an instability-correlated pathway would influence instability. Cell cycle pathways were negatively correlated with instability index (Table 2), and our instability prediction in tissues (Table 1) indicated intermediate levels of instability in many areas of the nervous system. This suggested that instability might be associated with the lack of mitotic activity in these tissues due to their high proportion of non-proliferating cells. Therefore, we tested directly whether cell cycle block would result in increased instability. To test this hypothesis, we took advantage of a clonal striatal cell line (ST*Hdh*^*Q*111/+^) derived from striatal primordia of *Hdh*^*Q*111/+ ^E14 embryos [30]. These cells divide at 33°C due to immortalization by the temperature sensitive SV40 large T antigen, but stop proliferating at 39°C due to the degradation of SV40 large T. We therefore compared the instability index of cells in cycling (33°C) and non-cycling (39°C) conditions over 9 weeks. As shown in Figure 7, the instability index increased over time only when the cells stopped cycling (+0.09 instability index units/week, linear regression model: Instability index ~Weeks, p value, 0.0015), but not when cells continuously proliferated (+5 × 10^-5 ^instability index units/week), consistent with the prediction from the negative correlation between cell cycle and the instability index. It is notable that the *HD *CAG repeat in ST*Hdh*^*Q*111 ^cells is extremely stable over multiple passages and under numerous different experimental conditions (data not shown). Cell cycle arrest is the only condition we have identified so far that has resulted in any expansion of the repeat. These findings indicate that the negative correlation of cell cycle pathways with the instability index more likely reflects a contribution of cell proliferation to preventing instability rather than a reduction of these pathways as a consequence of instability.

### Contribution of multiple processes to somatic instability

**Figure 8:**
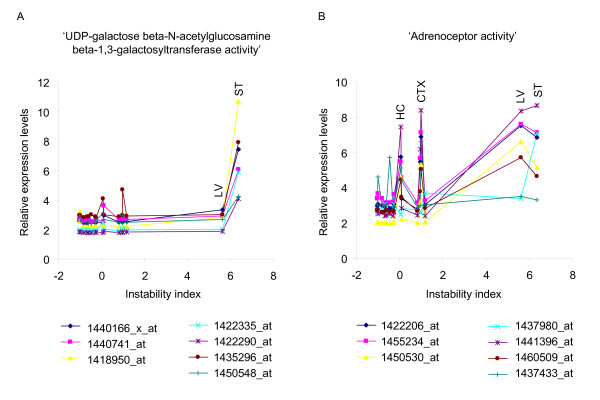
**Different combinations of many processes may be responsible for the different levels of somatic instability**. To investigate the levels of contributions from significant pathways in each tissue, we identified highly correlative probes (absolute Pearson coefficients > 0.6) in the two most significantly positively correlated gene sets, and plotted relative expression levels against measured instability indices in the 16 training set tissues. (A) 'UDP-galactose beta-N-acetylglucosamine beta-1,3-galactosyltransferase activity' gene set was the most significant pathway in the gene set analysis (positive correlation). This gene set is composed of 21 probes, and 7 probes were highly correlative (correlation coefficient > 0.6). The expression levels of these 7 probes in liver were low compared to those in striatum although instability indices are similar in these tissues. (B) 'Adrenoceptor activity' gene set was the second most significant pathway (positive correlation), and has 25 probes as members, of which 7 were highly correlative (correlation coefficient > 0.6). Interestingly, cortex or hippocampus showed similar expression levels of the highly correlative probes in this gene set to those in striatum, although instability indices in cortex or hippocampus were significantly lower than that of striatum. Graphs show 7 highly correlated probes for each gene set, and IDs are Affymetrix MG430 2.0 probe set IDs.

Although cell cycle pathways may be directly involved in modifying instability, some tissues (e.g. cerebellum) with a high proportion of non-proliferating cells were relatively stable. This indicated that each correlated pathway may explain a small part of the tissue instability and that the contributions of each pathway may be different for each tissue. Therefore, to investigate further the contributions of the different instability-correlated pathways, we compared the expression levels across different tissues of genes in the two most strongly correlated pathways (positive correlation). Interestingly, although 'UDP-galactose beta-N-acetylglucosamine beta-1,3-galactosyltransferase activity' was the most significantly correlated pathway (Table 2), liver which had a high instability index (5.6) showed a low level of gene expression in this pathway (Figure 8A). In addition, similar levels of gene expression in the 'adrenoceptor activity' pathway, the second most significantly correlated pathway (Table 2), occurred in hippocampus, cerebral cortex and striatum, with low (0.07), intermediate (0.98) and high (6.37) instability indices, respectively (Figure 8B). These results indicate that no single pathway can fully explain tissue-specific instability, strongly implying that somatic instability requires multiple processes that may be different in different tissues.

## Discussion

We have developed a novel approach for use in investigations of tissue-specific somatic *HD *CAG repeat instability that combines a reliable, high-throughput method for quantifying somatic instability with mathematical modeling based on gene expression data. Predictions based on our modeling were confirmed using genetic, biochemical and cell culture-based experiments, indicating the validity of our bioinformatics approach.

It has been proposed that somatic instability may be a consequence of disease pathogenesis [21], potentially explaining the striatal specificity of somatic expansion in HD. Our results directly demonstrate that HD pathogenesis does not explain the tissue specificity of *HD *CAG instability. In addition, DNA repair proteins have been found to be essential factors for somatic instability of trinucleotide repeats [7,8,11-16]. However, here we demonstrate that differences in expression levels of DNA repair genes do not underlie the tissue-specific differences in *HD *CAG instability. In addition, *Hdh *expression levels did not correlate with instability index in tissues (data not shown), confirming observations that although transcription through expanded repeats may be important in somatic instability [31], tissue-specific patterns are not reflected in the steady state levels of *Hdh *mRNA. Alternatively, our study suggests new pathways, notably metabolism, neurotransmitter, and cell cycle that may contribute, in combination, to the level of somatic instability in different tissues, providing a starting point to identify additional factors that contribute to somatic instability. Notably, there was no predominant factor that could explain the tissue-specificity of *HD *CAG instability, suggesting that patterns of instability are determined by the combined effects/interactions of many genes.

Somatic instability of trinucleotide repeats not only requires *trans*-acting factors, but has also been shown to depend on *cis*-acting sequences [10]. Thus, while certain tissues are more predisposed to somatic expansion, the expandability of a particular repeat in a particular tissue is further modified by its context. This could at least in part explain differences in the precise tissue patterns of somatic expansion in different diseases [2,29]. It would therefore be of interest to determine instability-correlated gene expression signatures and instability-correlated biological pathways for other trinucleotide repeat diseases. Instability-correlated genes/pathways that are shared between diseases would provide further insight into fundamental aspects of tissue-specific instability.

Our bioinformatics method based on gene expression data can only address aspects of tissue instability that are related to steady-state mRNA levels. In principle, however, a similar bioinformatic approach could be also applied to proteomics data. Irrespective of the particular method however, the strength of our approach is in its high-throughput, global and predictive nature, facilitating a number of important applications. Our GeneMapper quantification method is readily applicable to high-throughput assays such as screening small molecules that modulate instability in cells, or screening for genetic modifiers in mice. A powerful application of our bioinformatics approach is that the instability-correlated gene expression signature can be used as a surrogate marker for instability in situations where repeat instability cannot be directly measured. For example, gene expression databases can be screened to identify cell or tissue states that have the propensity for somatic instability, even in the absence of an expanded CAG repeat target as a read-out. Similarly, databases can be screened for compounds that reduce the instability propensity. Together, these approaches promise to accelerate the discovery of drugs that modulate instability and that are therefore candidate modifiers of disease.

## Conclusions

Our study demonstrates that multiple tissue factors including metabolism, neurotransmitter, and cell cycle combine to reflect the level of somatic instability in different tissues. Our findings also indicate that DNA repair proteins act largely in a non tissue-specific manner. In addition, the combination of our instability quantification method and mathematical modeling is a powerful strategy that has allowed us, in an unbiased manner, to gain critical new insights into the tissue specificity of trinucleotide repeat instability in HD. It opens the door to widespread downstream applications with the potential to make significant advances in novel avenues for therapeutic intervention in both Huntington's disease and trinucleotide expansion disorders in general.

## Methods

### Mice

*Hdh*^*Q*111 ^knock-in mice with 109 CAGs [18] were used for quantification of tissue instability and for microarray gene expression analyses (Affymetrix MG 430 2.0). Mice were genotyped as previously described [7]. For accelerated pathology models in cerebellum or striatum, *Hdh*^*Q*111/+ ^(CD1) and *Hdh*^*Q*92/+ ^mice (CD1) [6] were crossed with Harlequin (*Hq*) mutant (B6CBACa-A^W-J^/A) [23] and dopamine transporter (*DAT*) knockout mice (C57Bl/6J) [22], respectively. *Hdh*^*Q*92 ^mice were crossed with *DAT *knock-out mice and progeny intercrossed to generate *Hdh*^*Q*92^/^Q92 ^*DAT*^-/- ^mice and *Hdh*^*Q*92 ^*DAT*^+/+ ^control littermates for comparisons of instability. *Hdh*^*Q*111 ^males were crossed with *Hq/+ *females, and *Hdh*^*Q*111/+ ^*Hq/Y *males and control *Hdh*^*Q*111/+ ^*+/Y *littermate males used for comparisons of instability. All animal experiments were performed to minimize pain and discomfort, under an approved Institutional Animal Care and Use Committee protocol.

### CAG length determination and instability quantification

Genomic DNA, isolated from mouse tissues and cell lines (DNeasy, Qiagen), was used for PCR amplification using *HD *CAG repeat-specific primers as previously described [7]. The forward primer was fluorescently labeled with 6-FAM (Perkin Elmer) and PCR products were resolved using the ABI 3730 DNA analyzer (Applied Biosystems) using GeneMapper v.3.7 and GeneScan 500-LIZ as internal size standard to assign repeat size. GeneMapper traces were used to determine an instability index as described (Figure 1).

### Small pool-PCR

Genomic DNA was digested with *Eco*RV and diluted in 10 mM Tris-HCl, pH 8.0, 1 mM EDTA containing 0.1 μM carrier primer (MD16) to a final concentration of approximately 10 ng/μl. The amount of input DNA equivalent to a single amplifiable mutant *Hdh *allele was determined empirically using Poisson analysis, and for each tissue between 32 and 117 single mutant amplifiable molecules were analyzed. A nested PCR protocol was used, in which only the mutant (knock-in) *Hdh *allele is amplified. Mutant *Hdh *alleles were amplified using 0.5 μM MD16 primer 5'-CCCATTCATTGCCTTGCTGCTAAG (forward) [4] and 0.5 μM LKH5 primer 5'-TGGGTTGCTGGGTCACTCTGTC (reverse) [3] in 1× Thermo Scientific Custom PCR mix (containing 45 mM Tris-HCl pH 8.8, 11 mM ammonium sulfate, 4.5 mM MgCl_2_, 6.7 mM 2-mercaptoethanol, 4.4 μM EDTA, 1 mM dNTPs and 113 μg/ml BSA), 10% DMSO and 0.5 U units *Taq *polymerase (Fisher). Cycling conditions were 94°C 5 min, 35 cycles of 94°C 30 sec, 58°C 30 sec, 72°C 3 min, followed by 10 minutes at 72°C. PCR products were diluted 100-fold in TE and amplified in a second round using 0.8 μM Hu4 primer 5'-CCTGGAAAAGCTGATGAAGG (forward) and 0.8 μM Hu3 primer 5'-GGCGGCTGAGGAAGCTGAGGA (reverse) in a PCR buffer containing 67 mM Tris-HCl pH 8.8, 16.7 mM (NH_4_)_2_SO_4_, 2 mM MgCl2, 0.17 mg/mg BSA, 10 mM 2-mercaptoethanol, 10% DMSO, 200 μM dNTPs, with 0.5 U *Taq *polymerase (Fisher). Cycling conditions were 94°C 90 sec, 25 cycles of 94°C 30 sec, 65°C 30 sec, 72°C 90 sec, followed by 10 minutes at 72°. Hu4 was fluorescently labelled with 6-FAM (Applied Biosystems). PCR products were resolved using the ABI 3730 automated DNA analyzer (Applied Biosystems) using GeneMapper v.3.7 and GeneScan 500-LIZ as internal size standard to assign repeat size. *HD *CAG size was assigned as the highest peak. All PCR reactions were set up in a laminar flow hood and 20% of zero DNA control PCR reactions were included per run. To determine a small pool instability index we determined the frequency of each CAG repeat length, and multiplied each frequency by the number of repeats (+ or -) from the modal CAG length. These values were then summed.

### Analysis of GNF mouse Gene Expression Atlas and regression modeling

We used the mouse tissue gene expression database of Genomics Institute of the Novartis Research Foundation (mouse Gene Expression Atlas, GSE11339). All microarrays were background corrected and normalized by gcRMA. To identify an instability-correlated gene expression signature, Pearson correlation coefficients and corresponding p values between gene expression levels and instability indices of training samples (16 tissues, 2 gene expression replicates) were calculated for each probe, and the gene expression data was sorted by p values. We used Pearson correlation coefficients only as a ranking metric and this linear correlation information has not been used in actual modeling. Therefore, our models capture not just linear relationship but covariance between instability and expression. To identify an instability-correlated gene expression signature, we sequentially introduced the top n most highly correlated probes into the regression algorithms in a forward selection procedure, and calculated root mean squared error of prediction (RMSEP) by leave one out cross validation (LOO CV) of training samples (R, 2.4.1 and 'pls' package, 2.5.0). In addition to LOO CV of training samples, we further tested our model using 2 different test set samples. Firstly, we measured instability indices in additional tissues (muscle, olfactory bulb, white adipose tissue and adrenal gland (*Hdh*^*Q*111/+^, 5 months, n = 4-6 mice for each tissue) and compared them with instability indices predicted by our model. Secondly, we additionally analyzed gene expression profiles of striatum and cerebellum (*Hdh*^*Q*111/+^, 5 months, n = 1) and used these to predict instability indices for comparison to previously measured instability indices in these tissues. Test set RMSEP was calculated based on the difference between measured and predicted instability indices. Prediction of instability index for each of the tissues analyzed in mouse Gene Expression Atlas was based on our regression model and the instability-correlated signature.

### Gene set enrichment analysis

Using all probes, gene set enrichment analysis [27] was performed to sensitively identify significantly correlated pathways with instability index. Measured instability indices of training samples (16 tissues, Figure 2A) were used as continuous phenotype labels, and Pearson correlation was selected for a ranking metric. Our gene set database included pathways annotated by Gene Ontology, KEGG, GenMAPP, and the Molecular Signature Database from the Broad Institute. Significant gene sets were identified by permutation-based nominal p value (p < 0.01).

## Abbreviations

HD: Huntington's disease; *HD*: HD gene; SCAs: spinocerebellar ataxias; DM1: myotonic dystrophy type 1; PLSR: partial least square regression; LOO CV: leave-one-out cross validation.

## Competing interests

The authors declare that they have no competing interests.

## Authors' contributions

JL and VCW formulated the study and designed the experiments. JL, KK, ED, TG, and ETL performed experiments. JZ, AIS, JRW, TW, MB, and MC provided materials. JL, IK, JFG, MEM, and VCW wrote the paper. JFG, MEM, and VCW obtained funding to support the work. All authors read and approved the final manuscript.

## Supplementary Material

Additional file 1 **An instability-correlated gene expression signature**. List of 150 probes comprising instability-correlated gene expression signature. Click here for file

Additional file 2 **Correlations of DNA metabolism genes with instability index**. Genes potentially involved in DNA metabolism such as DNA synthesis and DNA repair were identified by the Gene Ontology biological process. Click here for file

Additional file 3 **Gene set enrichment analysis results of DNA metabolism pathways**. Gene set analysis of DNA metabolism pathways indicated that DNA metabolism gene sets were not significantly (p < 0.01) correlated with the instability index. Click here for file
