# Supplementary material for: A novel approach to investigate tissue-specific trinucleotide repeat instability
Source: BMC Syst Biol. 2010 Mar 19;4:29. doi: 10.1186/1752-0509-4-29 (PMC2856555; doi:10.1186/1752-0509-4-29)
Supplement: Additional file 3 — Gene set enrichment analysis results of DNA metabolism pathways. Gene set analysis of DNA metabolism pathways indicated that DNA metabolism gene sets were not significantly (p < 0.01) correlated with the instability index. [file 1752-0509-4-29-S3.DOC]

Supplementary Table 3. Gene set enrichment analysis results of DNA metabolism pathways. Gene set analysis of DNA metabolism pathways indicated that DNA metabolism gene sets were not significantly (p<0.01) correlated with the instability index.

| NAME | SIZE | NES | P value |
| --- | --- | --- | --- |
| BASE-EXCISION REPAIR | 28 | -1.31 | 0.2060 |
| DNA ALKYLATION | 60 | -0.70 | 0.9243 |
| DNA CATABOLISM | 17 | 1.25 | 0.2270 |
| DNA DAMAGE CHECKPOINT | 33 | -1.09 | 0.3758 |
| DNA DAMAGE RESPONSE, SIGNAL TRANSDUCTION | 52 | -1.15 | 0.3161 |
| DNA DAMAGE RESPONSE, SIGNAL TRANSDUCTION RESULTING IN INDUCTION OF APOPTOSIS | 20 | -1.13 | 0.3664 |
| DNA DAMAGE SIGNALING PATHWAY | 228 | -1.52 | 0.0323 |
| DNA HELICASE ACTIVITY | 18 | -0.99 | 0.4603 |
| DNA METHYLATION | 60 | -0.70 | 0.9243 |
| DNA MODIFICATION | 67 | -0.79 | 0.8376 |
| DNA PACKAGING | 411 | -1.12 | 0.3011 |
| DNA POLYMERASE | 53 | -1.55 | 0.0522 |
| DNA RECOMBINATION | 76 | -1.38 | 0.1486 |
| DNA REPAIR | 356 | -1.24 | 0.2373 |
| DNA REPLICATION | 251 | -1.53 | 0.0880 |
| DNA REPLICATION INITIATION | 28 | -1.57 | 0.0926 |
| DNA REPLICATION REACTOME | 89 | -1.73 | 0.0404 |
| DNA TOPOISOMERASE ACTIVITY | 19 | -1.33 | 0.1488 |
| DNA TOPOLOGICAL CHANGE | 22 | -1.27 | 0.1934 |
| DNA-DEPENDENT ATPASE ACTIVITY | 59 | -1.64 | 0.0638 |
| DNA-DEPENDENT DNA REPLICATION | 75 | -1.61 | 0.0640 |
| DNA-DIRECTED DNA POLYMERASE ACTIVITY | 53 | -1.58 | 0.0437 |
| DNA-DIRECTED RNA POLYMERASE ACTIVITY | 71 | -1.04 | 0.4133 |
| DNA-DIRECTED RNA POLYMERASE II, HOLOENZYME | 94 | -0.85 | 0.6284 |
| DNAFRAGMENTPATHWAY | 22 | -1.03 | 0.4593 |
| DNA-METHYLTRANSFERASE ACTIVITY | 15 | -1.20 | 0.2616 |
| DOUBLE-STRAND BREAK REPAIR | 15 | -0.74 | 0.7684 |
| GENOME STABILITY DNA REPAIR | 225 | -1.00 | 0.4599 |
| NUCLEOTIDE-EXCISION REPAIR | 50 | 0.66 | 0.9449 |
